# GNSS/eLoran Fusion-Based RAIM for Satellite-Deficient Environments

**DOI:** 10.3390/s26134295

**Published:** 2026-07-06

**Authors:** Jingling Li, Huabing Wu

**Affiliations:** 1 National Time Service Center, Chinese Academy of Sciences, Xi’an 710600, China; lijingling@ntsc.ac.cn; 2 University of Chinese Academy of Sciences, Beijing 100049, China; 3 Key Laboratory of Time Reference and Applications, Chinese Academy of Sciences, Xi’an 710600, China

**Keywords:** Receiver Autonomous Integrity Monitoring (RAIM), GNSS, eLoran, hybrid positioning, fault detection and identification, integrity enhancement, resilient PNT

## Abstract

Global Navigation Satellite Systems (GNSS) provide essential positioning, navigation, and timing (PNT) services for a wide range of safety-critical applications. However, GNSS performance degrades significantly in satellite-deficient or interference-prone environments. To address this limitation, this study proposes a hybrid GNSS/eLoran integrity monitoring framework based on a simplified Receiver Autonomous Integrity Monitoring (RAIM) architecture. In the proposed method, GNSS observations from satellite constellations and range-equivalent measurements from the enhanced Loran (eLoran) terrestrial system are jointly processed using a weighted least-squares estimator. Integrity monitoring is performed through a global chi-square consistency test combined with a solution separation strategy for fault identification and exclusion. Horizontal Protection Level (HPL) is derived from the covariance of the estimation process to ensure bounded positioning error under nominal and fault conditions. Unlike conventional GNSS-only RAIM, the proposed framework enables improved redundancy and fault observability in satellite-deficient scenarios by incorporating heterogeneous terrestrial measurements. Simulation experiments consider satellite faults, eLoran measurement disturbances, and inter-system clock bias effects. Results demonstrate that the proposed method maintains reliable fault detection capability and ensures that positioning errors remain consistently bounded by the protection level under all tested scenarios.

## 1. Introduction

Satellite navigation systems have become critical infrastructure in modern society, with wide-ranging applications across transportation, military operations, agriculture, surveying, and emergency rescue, among others [1]. By transmitting signals between satellites and receivers, these systems provide accurate positioning, navigation, and timing (PNT) services, substantially enhancing the efficiency and safety of various industries [2]. However, satellite navigation signals are not considered always reliable and may be subjected to a variety of interferences and faults [3]. For instance, signal quality is typically degraded by atmospheric disturbances, satellite malfunctions, or even deliberate jamming, thereby degrading the stability and accuracy of navigation services [4]. Ensuring the availability and reliability of satellite navigation services has, therefore, become a major challenge across many application domains.

To address this issue, Receiver Autonomous Integrity Monitoring (RAIM) technology has been developed [5]. RAIM enables real-time monitoring of received navigation signals and assesses whether they meet specified accuracy requirements. By comparing signals from multiple sources, RAIM can identify potential faults or anomalies and issue timely warnings, thereby ensuring the continuous and reliable operation of navigation systems. The application of RAIM technology has substantially enhanced the robustness and reliability of satellite navigation, especially under complex environmental conditions, and has become a key component of safety assurance in critical applications.

In general, the successful operation of RAIM requires two fundamental conditions: a minimum number of satellites and sufficient satellite geometry. When the minimum number of required satellites (typically five) is not available, a so-called RAIM hole occurs. Common RAIM algorithms primarily rely on snapshot methods that utilize only the current epoch’s pseudorange observations, including pseudorange comparison [6], least-squares residual method, and parity vector method [7]. These methods are capable of fault detection and identification using single-epoch observations. Yang et al. [8] proposed an improved method that dynamically allocates the missed detection rate based on the difficulty of satellite fault detection, along with a corresponding Vertical Protection Level (VPL) computation method. This approach minimizes the protection level without compromising overall integrity, thereby enhancing RAIM algorithm availability. Shu et al. introduced a weighted optimization approach based on satellite characteristic slopes to improve the sensitivity of fault detection in the presence of high-risk satellites. Wu et al. [9] proposed a RAIM enhancement method based on internal consistency precision, introducing internal precision thresholds to reduce missed detections and improve fault identification rates.

Joerger et al. [10] developed a new airborne RAIM algorithm, termed Unified RAIM (URAIM), which leverages both Direct RAIM (DRAIM) and Relative RAIM (RRAIM) [11]. They established a novel method for determining piecewise linear approximations of the optimal detection region in the parity space. As the alert limit requirements vary, the optimal detection boundary transitions between that obtained by traditional chi-square residual RAIM and solution separation (SS) RAIM. In [12], residual-based (RB) and (SS) RAIM methods were compared, and a parity-space representation for SS RAIM was developed.

Minimizing computational load often compromises accuracy, leading to unsampled RAIM holes and potential safety risks. Feng et al. [5] proposed a direct and computationally efficient method for predicting RAIM holes by precisely calculating satellite footprint boundaries, intersections, and analyzing topologies of overlap regions. Their method achieves high accuracy with minimal computational burden. Many RAIM implementations overlook precise terrain masking effects at specific locations, instead assuming a fixed elevation mask across the entire horizon. To address this, Radisic et al. [13] introduced a variable masking RAIM algorithm, demonstrating its practical utility for availability prediction in obstructed environments.

In addition to single-system RAIM, there has been increasing research into RAIM for fusion systems. Li et al. [14] introduced the IGG III weight function into GNSS/INS fusion navigation, proposing a RAIM model based on IGG III to detect gross errors. Khanafseh et al. [15] utilized GPS/INS Fusion navigation systems for spoofing detection by incorporating RAIM-based fault detection concepts, thereby tightening upper bounds on integrity risk. Bhatti et al. [16] proposed a novel algorithm based on the growth rate of typical test statistics, enabling earlier detection of slowly developing ramp faults and improving integrity performance. Crespillo et al. [17] analyzed a low-complexity coupling scheme using RAIM and velocity RAIM as core integrity systems, focusing particularly on the uncertainty propagation through strapdown inertial algorithms and its impact on protection levels.

Despite extensive development and successful deployment in aviation and safety-critical navigation, classical RAIM and even modern Advanced RAIM (ARAIM) frameworks are still fundamentally designed for homogeneous satellite ranging systems. Their statistical assumptions, fault models, and integrity risk allocation strategies are primarily built upon the characteristics of GNSS pseudorange measurements—namely, space-segment failures, independent measurement noise, and constellation-level fault hypotheses.

Classical RAIM methods are mainly based on single-epoch pseudorange consistency checks, including parity space methods, least-squares residual tests, and solution separation approaches. These methods assume homogeneous GNSS observations and independent Gaussian measurement noise. ARAIM extends classical RAIM by introducing multi-hypothesis fault modeling, integrity risk allocation, and protection level computation frameworks. However, ARAIM still fundamentally relies on GNSS constellation-based measurements and assumes satellite-centric fault modes. Recent studies have extended RAIM into multi-sensor fusion systems such as GNSS/inertial navigation system (INS) integration. These methods improve robustness by introducing external aiding information, but they remain within homogeneous or tightly coupled sensor architectures. However, none of these approaches explicitly address heterogeneous terrestrial-satellite hybrid ranging systems such as GNSS/eLoran with fundamentally different error structures and fault characteristics.

However, future resilient PNT architectures increasingly rely on heterogeneous ranging sources, where signals originate from fundamentally different physical infrastructures. Among these, enhanced Loran (eLoran) has re-emerged as a promising terrestrial complement to GNSS due to its high transmission power, low-frequency ground-wave propagation, and strong resistance to jamming and spoofing. Unlike satellite navigation signals, eLoran measurements are affected by propagation-dependent Additional Secondary Factors (ASF) [18], transmitter timing instabilities, and geographically correlated ground conductivity variations. These characteristics lead to error behaviors and fault modes that differ fundamentally from those of GNSS.

Existing GNSS-centric RAIM/ARAIM integrity models do not explicitly account for such heterogeneous measurement characteristics. In particular, three theoretical gaps remain insufficiently addressed: (1) Heterogeneous Error Statistics—eLoran measurement errors exhibit spatial correlation and environment-dependent biases, violating the independent and identically distributed assumptions commonly used in GNSS integrity monitoring. (2) Expanded Fault Hypothesis Space—In addition to satellite faults, a hybrid GNSS/eLoran system must consider terrestrial transmitter faults, regional ASF modeling errors, and potential inter-system timing biases. These introduce new integrity threat modes that are not represented in traditional ARAIM fault trees. (3) Integrity Risk Reallocation—The inclusion of non-satellite ranging sources changes the structure and probability distribution of fault hypotheses, requiring a re-allocation of integrity risk among satellite and terrestrial fault events.

Therefore, simply treating eLoran measurements as additional “satellite-like” observations within a conventional RAIM framework is insufficient from an integrity-theoretic perspective. A dedicated extension of the integrity model is required to properly characterize heterogeneous measurement statistics and expanded fault modes.

To address these challenges, this paper proposes a hybrid GNSS/eLoran integrity monitoring framework inspired by integrity monitoring concepts developed for RAIM. The main focus of this study is the development and validation of a single-measurement fault detection and exclusion scheme under heterogeneous observation conditions.

The implemented model considers GNSS and eLoran measurement faults as independent single-fault hypotheses and evaluates system performance using solution separation and chi-square-based consistency checking.

Although the overall framework is designed to be extensible to broader fault scenarios, including transmitter faults, propagation-related ASF anomalies, and inter-system timing biases, these extensions are not fully incorporated into the current implementation. Instead, representative cases such as eLoran measurement disturbances and inter-system clock bias effects are included in the experimental section to validate the robustness of the proposed method under heterogeneous error conditions. Therefore, the present work should be regarded as a first-step extension of RAIM to heterogeneous GNSS/eLoran ranging systems rather than a complete integrity framework covering all possible fault modes.

By extending RAIM concepts originally developed for satellite-only systems to a heterogeneous ranging framework, this work provides a theoretical foundation for integrity monitoring in future resilient PNT architectures that integrate space-based and terrestrial navigation systems.

The remainder of this paper is organized as follows. Section 2 introduces the hybrid GNSS/eLoran observation model and the associated stochastic error characteristics. Section 3 presents the proposed integrity monitoring framework, including the weighted least-squares estimation, global consistency test, and solution separation-based fault identification strategy. Section 4 describes the simulation setup and experimental scenarios, including satellite-deficient configurations and representative fault cases. Section 5 presents and discusses the experimental results in terms of fault detection performance and protection level evaluation. Finally, Section 6 summarizes the main findings and outlines potential directions for future work.

## 2. Hybrid Observation Model and Integrity-Oriented Measurement Representation

### 2.1. Role of eLoran in an Integrity Framework

The proposed system integrates GNSS and eLoran ranging sources within an integrity-monitoring framework. Unlike GNSS, which relies on spaceborne transmitters, eLoran is a terrestrial low-frequency system characterized by high transmission power and strong resistance to jamming and spoofing. Because of its fundamentally different signal propagation mechanism, eLoran is not simply treated as a low-accuracy “satellite-like” source, but as a heterogeneous ranging modality whose statistical properties and fault behaviors must be explicitly modeled in the integrity framework.

In this study, Earth Observation (EO) data are used only as auxiliary information for environmental corrections and stochastic characterization. EO does not serve as an independent positioning measurement but provides: (1) bias corrections for ionospheric delay, tropospheric delay, terrain masking, and eLoran ASF variations; (2) uncertainty characterization, allowing the measurement covariance to be adaptively inflated under adverse environmental conditions.

Thus, EO information modifies the observation and noise models but does not introduce additional states into the positioning solution.

### 2.2. Fused Observation Equation

Because the positioning accuracy of eLoran is inherently lower than that of GNSS, it is necessary to enhance the precision of eLoran signals before fusion [19]. Two commonly adopted approaches are: (1) applying corrections derived from an external high-accuracy reference source, such as an auxiliary GNSS receiver [20], and (2) utilizing differential Loran (DLoran) corrections to compensate for signal propagation delays [21].

In addition, the clock offset between the eLoran and GNSS must be addressed. Similarly to the above, this offset can be corrected either by synchronizing both systems to an external high-precision time reference (e.g., an atomic clock) or by employing counters to estimate and correct the relative clock bias between the GNSS and eLoran receivers.

To enable the fusion of eLoran and BeiDou Navigation Satellite System (BDS) observations, it is necessary to unify their coordinate systems [22]. The World Geodetic System 1984 (WGS 84) geodetic coordinates employed by eLoran must be converted into the Earth-Centered Earth-Fixed (ECEF) coordinates used by BDS. Coordinate conversions follow the standard WGS-84 to ECEF transformation.

The GNSS pseudorange model can be expressed as follows [4]:

(1) $$\rho_{i}=\left\|x_{pos}-s_{i}\right\|+c\Delta t+d_{i}^{iono}+d^{tropo}+\varepsilon_{i}^{GNSS}$$

where the $d_{i}^{iono}$ and $d^{tropo}$ terms can be corrected or parameterized using EO data (or ionospheric models and numerical weather models, numerical weather models).

The linearized form is:

(2) $$\Delta \rho_{i}\approx h_{i}^{T}\Delta x+\varepsilon_{i}^{GNSS}$$

where $h_{i}={\left[\frac{{\left(x_{0}-s_{i}\right)}^{T}}{∥x_{0}-s_{i}∥},1\right]}^{T}$, $\rho_{i}$ is the pseudorange measurement from the i-th GNSS satellite; $x_{pos}$ and $s_{i}$ are the receiver and satellite position vectors in the Earth-Centered Earth-Fixed coordinate frame, respectively; c is the speed of light; Δt is the receiver clock offset expressed in seconds; $d_{i}^{\mathrm{iono}}$ and $d_{i}^{\mathrm{tropo}}$ are the ionospheric and tropospheric delay terms, respectively; and $\varepsilon_{i}^{\mathrm{GNSS}}$ denotes the remaining GNSS measurement error.

In the linearized model, $\Delta \rho_{i}$ is the observed-minus-computed pseudorange residual, $\Delta x={\left[\Delta x_{r},\Delta y_{r},\Delta z_{r},c\Delta t\right]}^{T}$ is the four-dimensional state correction vector, and $h_{i}$ is the corresponding design vector evaluated at the approximate receiver position $x_{0}$. The first three elements of $h_{i}$ represent the geometric line-of-sight derivatives, while its last element corresponds to the receiver clock-bias state.

The eLoran pseudorange model is expressed as:

(3) $$p_{j}=c(t_{rx}-t_{tx,j})+ASF_{j}(x_{pos})+\varepsilon_{j}^{L}$$

where ASF represents the groundwave propagation delay and terrain effects, which can be modeled as parameters or scenario-dependent functions with the assistance of EO.

Linearized:

(4) $$\Delta p_{j}\approx g_{j}^{T}\Delta x+\frac{\partial ASF_{j}}{\partial x}\Delta x+\varepsilon_{j}^{L}$$

where $p_{j}$ is the range-equivalent eLoran measurement associated with the j-th transmitter; $t_{\mathrm{rx}}$ is the signal reception time; $t_{\mathrm{tx},j}$ is the transmission time of the j-th eLoran transmitter; ${\mathrm{ASF}}_{j}(x_{\mathrm{pos}})$ is the location-dependent ASF correction; and $\varepsilon_{j}^{L}$ denotes the remaining eLoran measurement error.

In Equation (5), $\Delta p_{j}$ is the linearized eLoran range residual, $g_{j}$ is the geometric design vector associated with the receiver-to-transmitter range, and $\partial {\mathrm{ASF}}_{j}/\partial x$ is the spatial gradient of the ASF correction evaluated at the approximate receiver position. The term $(\partial {\mathrm{ASF}}_{j}/\partial x)\Delta x$, therefore, represents the first-order variation in the ASF correction caused by a change in receiver position.

To enable integrity monitoring under satellite-deficient conditions, GNSS and eLoran measurements are integrated at the observation level. Because the two systems have different signal propagation mechanisms, clock references, measurement accuracies, and error characteristics, the hybrid observation model should explicitly distinguish between GNSS pseudorange observations and eLoran range-equivalent observations.

In this study, environmental information is used as auxiliary correction information rather than as an independent positioning observation. It is introduced to support empirical compensation and uncertainty characterization of atmospheric delay, terrain-related effects, and eLoran propagation errors. Therefore, no independent EO vector is included in the fused observation equation. Instead, environmental correction uncertainty is reflected in the corrected residuals and in the measurement covariance matrix.

Let *m*_*G*_ denote the number of available GNSS pseudorange observations and *m*_*L*_ denote the number of available eLoran observations. Before inter-system clock processing, the full receiver state correction vector is written as:

(5) $$\Delta x_{0}={\left[\Delta x_{r}\;\Delta y_{r}\;\Delta z_{r}\;c\Delta t_{G}\;c\Delta t_{L}\right]}^{T}$$

where Δ*x*_*r*_, Δ*y*_*r*_, and Δ*z*_*r*_ are the receiver position corrections in the Earth-Centered Earth-Fixed frame, *c*Δ*t*_*G*_ is the GNSS receiver clock bias expressed in meters, and *c*Δ*t*_*L*_ is the eLoran receiver clock bias expressed in meters.

Because GNSS and eLoran observations are referenced to different timing chains, their relative time offset must be handled before forming the final hybrid solution. The eLoran clock term can be decomposed as:

(6) $$c\Delta t_{L}=c\Delta t_{G}+c\Delta t_{LG}$$

where *c*Δ*t*_*LG*_ denotes the inter-system time offset between the GNSS and eLoran measurement domains. In practical implementation, this offset can be calibrated using an external time reference, counter-based time-transfer processing, or an estimated inter-system bias correction. After this correction, the residual inter-system time offset is either compensated in the eLoran observation correction term or treated as a bounded residual error in the covariance model. Therefore, the state vector used in the subsequent weighted least-squares estimation can be reduced to:

(7) $$\Delta x_{0}={\left[\Delta x_{r}\;\Delta y_{r}\;\Delta z_{r}\;c\Delta t\right]}^{T}$$

where cΔt represents the equivalent common receiver clock bias after GNSS/eLoran clock alignment. This treatment preserves the physical distinction between the two original clock biases while allowing the fused positioning model to be expressed in a standard four-state form.

In the hybrid GNSS/eLoran observation model, an inter-system time offset exists due to the independent timing realization of GNSS and eLoran systems. In the implementation, this bias is not explicitly estimated as an independent state variable in the final navigation solution. Instead, it is first mitigated through a calibration procedure using time-aligned reference data (e.g., receiver-based synchronization or external time reference), which reduces the nominal offset between the two systems:

(8) $$\sigma_{L,j}^{2}\leftarrow \sigma_{L,j}^{2}+\sigma_{clk}^{2}$$

where $\sigma_{clk}^{2}$ represents the residual uncertainty of the GNSS/eLoran time alignment after calibration.

From an integrity perspective, this residual term propagates through the weighted least-squares estimation and directly contributes to both the residual vector and the covariance matrix used in Horizontal Protection Level (HPL) computation. Therefore, the inter-system clock offset is implicitly included in the integrity monitoring process through its stochastic contribution rather than as an explicit fault hypothesis.

After calibration, a residual inter-system timing error remains. This residual bias is modeled as a bounded stochastic term and incorporated into the measurement noise model rather than treated as a deterministic correction. Specifically, its effect is absorbed into the eLoran variance component:

For the *i*-th GNSS satellite, the corrected pseudorange residual is defined as:

(9) $$y_{G,i}=\rho_{G,i}^{obs}-{\hat{\rho }}_{G,i}-{\hat{d}}_{ion,i}-{\hat{d}}_{trop,i}-{\hat{d}}_{sat,i}-{\hat{d}}_{rel,i}$$

where $\rho_{G,i}^{obs}$ is the observed GNSS pseudorange, ${\hat{\rho }}_{G,i}$ is the computed geometric range between the approximate receiver position and the satellite position, ${\hat{d}}_{ion,i}$ and ${\hat{d}}_{trop,i}$ are the ionospheric and tropospheric delay corrections, respectively, ${\hat{d}}_{sat,i}$ denotes satellite orbit and clock corrections, and ${\hat{d}}_{rel,i}$ represents relativistic and other minor corrections.

After linearization around the approximate receiver position, the GNSS residual model is:

(10) $$y_{G,i}=h_{G,i}^{T}\cdot \Delta x+v_{G,i}$$

with:

(11) $$h_{G,i}^{T}=\left[\begin{array}{cc} -u_{G,i}^{T} & 1 \end{array}\right]$$

where $u_{G,i}$ is the line-of-sight unit vector from the receiver to the *i*-th GNSS satellite, and $v_{G,i}$ is the residual GNSS measurement error after applying deterministic corrections.

For the *j*-th eLoran transmitter, the corrected eLoran range-equivalent residual is expressed as:

(12) $$y_{L,j}=p_{L,j}^{obs}-{\hat{p}}_{L,j}-{\hat{PF}}_{j}-{\hat{SF}}_{j}-{\hat{ASF}}_{j}-{\hat{b}}_{LG}$$

where $p_{L,j}^{obs}$ is the observed eLoran range-equivalent measurement, ${\hat{p}}_{L,j}$ is the computed geometric distance between the receiver and the *j*-th eLoran transmitter, ${\hat{PF}}_{j}$ is the primary factor correction, ${\hat{SF}}_{j}$ is the secondary factor correction, ${\hat{ASF}}_{j}$ is the ASF correction, and ${\hat{b}}_{LG}$ is the estimated or calibrated GNSS/eLoran inter-system bias correction.

The ASF term accounts for the propagation delay caused by ground conductivity, terrain, coastal transition, and other environment-dependent effects. In this study, ASF-related corrections are treated empirically, and the remaining ASF uncertainty is represented in the eLoran measurement variance.

After linearization, the eLoran residual model is:

(13) $$y_{L,j}=h_{L,j}^{T}\cdot \Delta x+v_{L,j}$$

where:

(14) $$h_{L,j}^{T}=\left[-u_{L,j}^{T}\;1\right]$$

Here, $u_{L,j}$ is the unit vector from the receiver to the *j*-th eLoran transmitter, and $v_{L,j}$ denotes the residual eLoran measurement error after applying propagation and inter-system bias corrections. Compared with GNSS residual errors, eLoran residual errors usually contain larger environment-dependent components, especially those caused by imperfect ASF compensation.

By stacking all GNSS and eLoran residuals, the hybrid linearized observation model is obtained as:

(15) $$y=\left[\begin{array}{l} y_{G}\\ y_{L} \end{array}\right]=\left[\begin{array}{l} h_{G}\\ h_{L} \end{array}\right]\Delta x+\left[\begin{array}{l} v_{G}\\ v_{L} \end{array}\right]$$

or equivalently:

(16) y=HΔx+v,v∼N(0,R)

where $y\in {\mathbb{R}}^{m_{G}+m_{L}}$ is the hybrid measurement residual vector, $H\in {\mathbb{R}}^{(m_{G}+m_{L})\times 4}$ is the hybrid design matrix, $x\in {\mathbb{R}}^{4}$ is the reduced state correction vector after clock alignment, and v is the hybrid residual noise vector.

The hybrid measurement covariance matrix is written as:

(17) $$R=\left[\begin{array}{cc} R_{G} & 0\\ 0 & R_{L} \end{array}\right]$$

The off-diagonal blocks between GNSS and eLoran are set to zero because the residual GNSS pseudorange errors and eLoran propagation errors are assumed to be statistically independent after correction. The GNSS covariance matrix is modeled as:

(18) $$R_{G}=\mathrm{diag}\left(\sigma_{G,1}^{2},\sigma_{G,2}^{2},\ldots ,\sigma_{G,m_{G}}^{2}\right)$$

where $\sigma_{G,i}^{2}$ includes receiver noise, multipath, residual atmospheric delay, satellite orbit and clock uncertainty, and other unmodeled GNSS errors. An elevation-dependent form may be used as:

(19) $$\sigma_{G,i}^{2}=\sigma_{0,G}^{2}+\frac{\sigma_{el,G}^{2}}{\sin^{2}E_{i}}$$

where *E*_*i*_ is the elevation angle of the *i*-th satellite.

For eLoran measurements, the covariance matrix is modeled using a conservative variance-inflation approach:

(20) $$R_{L}=\mathrm{diag}\left(\sigma_{L,1}^{2},\sigma_{L,2}^{2},\ldots ,\sigma_{L,m_{L}}^{2}\right)$$

where the variance of the *j*-th eLoran measurement is expressed as:

(21) $$\sigma_{L,j}^{2}=\sigma_{rx,L,j}^{2}+\sigma_{tx,L,j}^{2}+\sigma_{PF,j}^{2}+\sigma_{SF,j}^{2}+\sigma_{ASF,j}^{2}+\sigma_{LG}^{2}$$

Here, $\sigma_{rx,L,j}^{2}$ is the receiver noise variance, $\sigma_{tx,L,j}^{2}$ is the transmitter timing uncertainty, $\sigma_{PF,j}^{2}$, $\sigma_{SF,j}^{2}$, and $\sigma_{ASF,j}^{2}$ denote the residual uncertainties associated with the primary factor, secondary factor, and ASF corrections, respectively, and $\sigma_{LG}^{2}$ represents the residual uncertainty of the GNSS/eLoran inter-system bias after calibration.

Although this study adopts the diagonal variance-inflation model in Equation (19), a more general eLoran covariance model can include spatial correlation among ASF residuals:

(22) $$R_{L}=D_{L}+\Sigma_{ASF}$$

where $D_{L}$ is the diagonal independent-noise component and $\sum_{ASF}$ represents the spatially correlated ASF uncertainty. The elements of $\sum_{ASF}$ may be expressed as:

(23) $${\left[\sum_{ASF}\right]}_{jk}=\rho_{jk}\sigma_{ASF,j}\sigma_{ASF,k},\;0\leq \rho_{jk}\leq 1$$

where *ρ*_*jk*_ is the correlation coefficient between the residual ASF errors of the *j*-th and *k*-th eLoran propagation paths. In the absence of reliable ASF correlation information, the diagonal model in Equation (20) is adopted to maintain consistency with the implemented algorithm. The correlated model in Equation (22) provides a possible extension for future integrity analysis when spatial ASF correlation statistics are available [23,24].

In this formulation, the contribution of each measurement to the fused solution is determined by its corresponding covariance. GNSS observations usually receive larger weights because of their smaller residual variances, while eLoran observations provide additional geometric diversity and measurement redundancy. This is particularly important when the number of visible satellites is insufficient for conventional RAIM fault detection and exclusion.

### 2.3. EO-Assisted Correction and Uncertainty Representation

Environmental information is used as auxiliary correction information rather than as an independent positioning observation. For GNSS measurements, it supports the correction and uncertainty characterization of residual atmospheric delay, multipath, and site-dependent effects. For eLoran measurements, it is mainly used to characterize propagation-related errors, particularly residual ASF effects associated with ground conductivity, terrain, coastal transitions, and seasonal variations. The remaining environmental uncertainty is incorporated into the measurement covariance matrix, preventing excessive weighting of eLoran observations under uncertain propagation conditions.

The implemented model adopts a diagonal variance-inflation approach for computational tractability. Although residual ASF errors may be spatially correlated across propagation paths, the corresponding off-diagonal covariance terms are not included in the present implementation. This approximation may slightly overestimate effective measurement redundancy; a spatially correlated ASF covariance model will, therefore, be considered when reliable regional statistics become available.

To maintain computational tractability, the covariance of eLoran measurements is modeled as a diagonal matrix with variance inflation:

(24) $$R_{L}=\mathrm{diag}(\sigma_{ASF,j}^{2}+\sigma_{tx,j}^{2}+\sigma_{rx}^{2})$$

where $\sigma_{ASF,j}^{2}$ represents residual ASF uncertainty after correction, $\sigma_{tx,j}^{2}$ denotes transmitter timing uncertainty, and $\sigma_{rx}^{2}$ denotes receiver noise variance.

Although ASF errors may exhibit spatial correlation across propagation paths, the present study neglects off-diagonal correlation terms:

(25) $$R_{L}^{corr}=R_{L}+\Sigma_{ASF}^{off}$$

where $\Sigma_{ASF}^{off}$ denotes the neglected cross-correlation component.

This simplification may lead to a slightly optimistic estimation of detection performance, as correlated ASF errors can reduce effective measurement redundancy. However, since the proposed integrity framework is designed as a conservative RAIM-type detector with inflated variance modeling, the diagonal approximation remains appropriate for feasibility validation under satellite-deficient scenarios. Neglecting the spatial correlation of ASF residuals may lead to a slight overestimation of effective measurement redundancy and consequently somewhat optimistic fault-detection performance.

## 3. Proposed GNSS/eLoran Integrity Monitoring Framework

The objective of the proposed hybrid RAIM algorithm [25,26] is to detect and exclude faulty measurements in satellite-deficient environments by using the redundant information provided by both GNSS and eLoran observations. Based on the hybrid observation model established in Section 2, the corrected measurement residual vector is expressed in Equation (15).

The weighted least-squares solution is obtained as:

(26) $$\Delta \hat{x}={\left(H^{T}WH\right)}^{-1}H^{T}Wy$$

where the weight matrix is defined as:

(27) $$W=R^{-1}$$

Thus, each GNSS or eLoran measurement is weighted according to its residual uncertainty. Measurements with smaller variance contribute more strongly to the position solution, while measurements with larger uncertainty are down-weighted.

The post-fit residual vector is:

(28) $$r=y-H\Delta \hat{x}$$

A global consistency test is then constructed using the weighted residual norm:

(29) $$T=r^{T}Wr$$

Under the fault-free hypothesis *H*_0_, the statistic *T* approximately follows a chi-square distribution with *m* − *n* degrees of freedom, where *m* is the total number of GNSS and eLoran measurements and *n* is the number of estimated states:

(30) $$T\sim \chi_{m-n}^{2}$$

The detection threshold is determined by the specified false alarm probability *P*_*FA*_:

(31) $$T_{D}=\chi_{m-n}^{2^{-1}}(1-P_{FA})$$

If:

(32) $$T\leq T_{D}$$

the measurement set is considered consistent and no fault is declared. Otherwise, a fault alarm is triggered and the fault identification and exclusion procedure are performed.

### 3.1. Fault Hypothesis Definition

In the hybrid GNSS/eLoran system, faults may originate from either GNSS or eLoran measurements. Therefore, the faulted observation model under the *k*-th fault hypothesis is written as:

(33) $$H_{k}:y=H\Delta x+F_{k}f_{k}+v$$

where *F*_*k*_ is the fault selection matrix and *f*_*k*_ is the corresponding fault magnitude vector. For a single-measurement fault, *F*_*k*_ reduces to a unit selection vector that identifies the suspected faulty GNSS satellite or eLoran transmitter.

The considered fault hypotheses include:

(34) $$H=\{H_{G,1},\ldots ,H_{G,m_{G}},H_{L,1},\ldots ,H_{L,m_{L}}\}$$

where *H*_*G*,*i*_ denotes a fault in the *i*-th GNSS measurement and *H*_*L*,*j*_ denotes a fault in the *j*-th eLoran measurement. This formulation allows the same RAIM framework to handle both satellite faults and eLoran measurement faults.

### 3.2. Fault Identification and Exclusion

Once the global test statistic exceeds the detection threshold, each candidate measurement is tested by temporarily excluding it from the hybrid measurement set. For the k-th exclusion hypothesis, the corresponding measurement vector, design matrix, and covariance matrix are denoted as $y^{\left(k\right)}$, $H^{\left(k\right)}$ and $R^{\left(k\right)}$, respectively.

The state estimate after excluding the k-th measurement is:

(35) $$\Delta {\hat{x}}^{\left(k\right)}={\left[{\left(H^{\left(k\right)}\right)}^{T}{\left(R^{\left(k\right)}\right)}^{-1}H^{\left(k\right)}\right]}^{-1}{\left(H^{\left(k\right)}\right)}^{T}{\left(R^{\left(k\right)}\right)}^{-1}y^{\left(k\right)}$$

The corresponding residual test statistic is:

(36) $$T^{\left(k\right)}={\left(r^{\left(k\right)}\right)}^{T}{\left(R^{\left(k\right)}\right)}^{-1}r^{\left(k\right)}$$

The measurement whose exclusion produces the smallest residual statistic is identified as the most likely faulty measurement:

(37) $$k^{*}=\arg^{*}min_{k}T^{\left(k\right)}.$$

If $T^{\left(k^{*}\right)}$ falls below the detection threshold, the $k^{*}$-th measurement is excluded and the navigation solution is recomputed using the remaining measurements. Otherwise, the fault is regarded as not reliably isolatable, and the system declares that fault exclusion is unavailable.

### 3.3. Horizontal Protection Level

Integrity protection is quantified using the covariance of the horizontal position estimate:

(38) $$P_{h}=T_{h}PT_{h}^{T}$$

The Horizontal Protection Level is defined as:

(39) $$HPL=K\cdot \sqrt{trace(P_{h})}$$

where *K* is derived from the required integrity risk bound.

The system guarantees:

(40) HPE≤HPL

under nominal and correctly identified fault conditions.

### 3.4. Integrity Decision Logic

The proposed integrity monitoring framework operates in two modes:

Nominal mode: no fault detected ($T\leq T_{th}$);

Fault mode: global alert triggered ($T>T_{th}$).

In fault mode, the system attempts solution separation-based identification. If isolation is successful, the faulty measurement is excluded and the solution is updated. Otherwise, a conservative alert is issued to preserve safety integrity.

To ensure consistency in integrity evaluation, the HPL is used as the integrity bound for position errors, while the Minimum Detectable Bias (MDB) characterizes the smallest fault magnitude that triggers detection under the chi-square test.

MDB is determined by the detection threshold corresponding to a 0.95 confidence level of the global test statistic:

(41) $$T=v^{T}R^{-1}v\sim \chi^{2}(m-n)$$

Detection, missed detection, and false alarm rates are evaluated based on this threshold in the time domain.

The 0.95 confidence level provides a standard trade-off between detection sensitivity and false alarm robustness in snapshot RAIM-type integrity monitoring.

### 3.5. Complexity Analysis

The computational complexity of the proposed hybrid RAIM algorithm is dominated by the weighted least-squares (WLS) estimation and the solution separation procedure.

Let m denote the total number of GNSS and eLoran observations, and n denote the number of estimated states. The WLS solution requires the inversion of an n × n normal matrix, resulting in a computational complexity of $O(n^{3})$. Since n = 4 in the proposed model, this step is constant time in practice.

The global test statistic requires residual computation with complexity $O\left(m\right)$. For fault identification and exclusion, a solution separation strategy is applied, which requires solving (m) reduced WLS problems, leading to a worst-case complexity of $O(m\cdot n^{3})$. Therefore, the overall complexity per epoch can be approximated as: $O(m\cdot n^{3})$.

Given that n is fixed and small, the algorithm scales approximately linearly with the number of observations. The computational complexity of the proposed algorithm is comparable to that of conventional solution-separation RAIM and substantially lower than that of full multi-hypothesis ARAIM.

Table 1 summarizes the principal differences between representative existing methods and the proposed framework.

Compared with ARAIM, the proposed method avoids multi-hypothesis probability propagation, thereby reducing computational burden.

**Figure 1:**
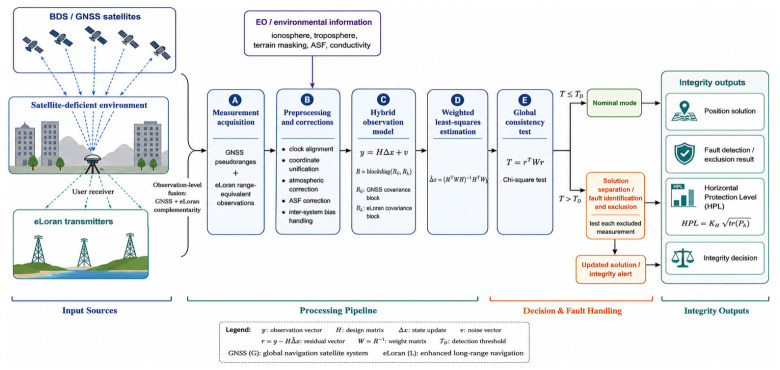
System architecture of the proposed GNSS/eLoran hybrid RAIM framework.

Figure 1 illustrates the overall architecture of the proposed GNSS/eLoran hybrid RAIM framework. GNSS pseudoranges and eLoran range-equivalent measurements are first collected and corrected using clock alignment, coordinate transformation, atmospheric compensation, ASF correction, and inter-system bias handling. The corrected observations are then integrated through a hybrid observation model and processed using weighted least-squares estimation. A global chi-square consistency test is subsequently applied for fault detection. When a fault is detected, solution separation is performed to identify and exclude the faulty measurement. The final outputs include the position solution, fault detection and exclusion results, the Horizontal Protection Level, and the corresponding integrity decision.

## 4. Results

**Figure 2:**
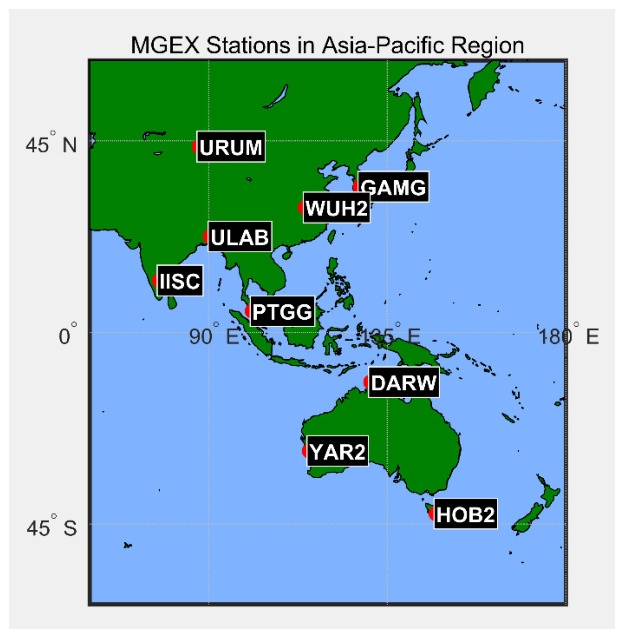
Distribution of MGEX stations in the Asia–Pacific region.

Considering the comprehensive coverage of BDS in the Asia–Pacific region, especially the availability of IGSO satellites, the experimental region was selected within this area. The distribution of the selected Multi-GNSS Experiment (MGEX) stations is shown in Figure 2. Data from multiple stations were used in the experiments, and the results obtained at station WUH2 are mainly presented for detailed analysis. The GNSS observations were generated from IGS precise orbit and clock products and processed using a custom MATLAB R2024a-based simulation framework. The positioning and integrity algorithms were independently verified by reproducing standard least-squares positioning results and comparing the obtained residual statistics with theoretical expectations.

To evaluate the integrity-monitoring capability of the proposed GNSS/eLoran hybrid RAIM algorithm, artificial faults were injected into satellite SAT3. A total of 1440 epochs were used in the experiment. The fault interval was set from epoch 480 to epoch 960, while epochs 1–479 and 961–1440 were treated as fault-free intervals. Two representative fault modes were considered: a step fault and a ramp fault. The step fault represents an abrupt pseudorange anomaly, such as that caused by hardware failure or sudden signal interference, while the ramp fault represents a slowly developing measurement bias. The chi-square test confidence level was set to 0.95. Unless otherwise stated, the injected faults were applied only to SAT3.

The experimental settings are summarized in Table 2. Satellite subsets were selected by minimizing the geometric dilution of precision (GDOP), while eLoran observations were generated using stations from the East China Sea chain. GNSS and eLoran measurements were assigned system-specific stochastic models. A 20 m step bias and a 0.05 m/s ramp bias were injected to represent abrupt and gradually developing faults, respectively.

### 4.1. Fault Detection Performance

First, twelve BDS satellites and three eLoran stations were fused to evaluate the fault-detection and fault-identification performance of the proposed algorithm under high-redundancy observation conditions.

**Figure 3:**
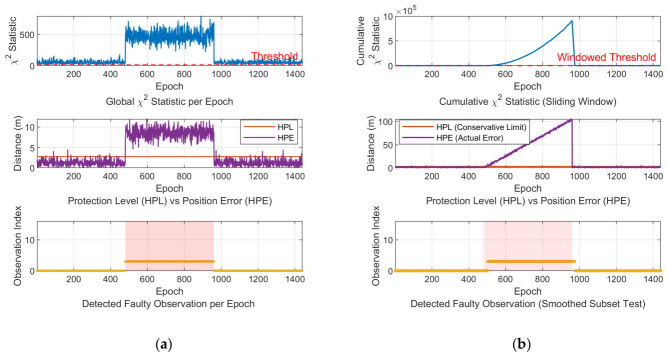
Detection and identification results for the 12-satellite + 3-eLoran configuration under (**a**) a 20 m step fault and (**b**) a 0.05 m/s ramp fault. The top, middle, and bottom panels show the chi-square statistic, the HPL/Horizontal Position Error (HPE) comparison, and the identified faulty observation, respectively.

**Figure 4:**
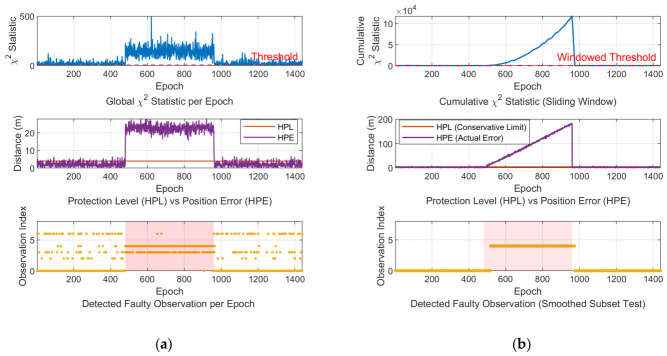
Detection and identification results for the 3-satellite + 3-eLoran configuration under (**a**) a 20 m step fault and (**b**) a 0.05 m/s ramp fault. The top, middle, and bottom panels show the chi-square statistic, the HPL/HPE comparison, and the identified faulty observation, respectively.

**Figure 5:**
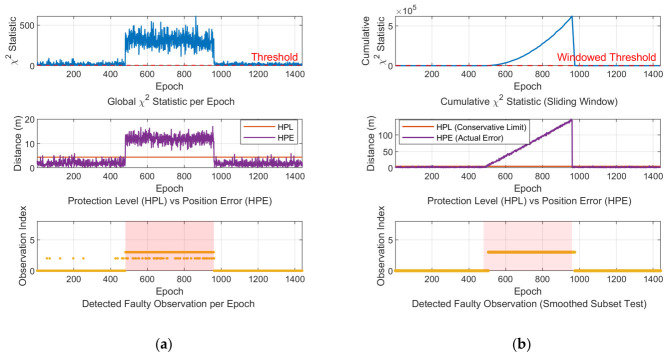
Detection and identification results for the 5-satellite + 1-eLoran configuration under (**a**) a 20 m step fault and (**b**) a 0.05 m/s ramp fault. The top, middle, and bottom panels show the chi-square statistic, the HPL/HPE comparison, and the identified faulty observation, respectively.

Figure 3, Figure 4 and Figure 5 compare the fault-detection performance of the 12-satellite + 3-eLoran, 3-satellite + 3-eLoran, and 5-satellite + 1-eLoran configurations under step- and ramp-fault conditions. All three configurations exhibit similar temporal patterns because they use the same observation epochs, fault interval, injected fault models, and detection logic. Step faults produce an abrupt increase in the global test statistic and are detected rapidly, whereas ramp faults generate a gradual response and are more difficult to distinguish from nominal measurement noise during the early stage of fault growth.

Despite their similar curve shapes, the three configurations differ substantially in response magnitude, threshold separation, position-error variation, and fault-identification continuity. The 12-satellite + 3-eLoran configuration provides the strongest and most stable detection response because of its high measurement redundancy and favorable geometry. The 3-satellite + 3-eLoran configuration shows the weakest response and greater detection variability, but it still maintains practical fault-monitoring capability under severe satellite deficiency. The 5-satellite + 1-eLoran configuration achieves intermediate performance and is generally more stable than the 3-satellite + 3-eLoran configuration, although both contain six observations. This result indicates that detection performance depends not only on the total number of observations but also on their composition, geometry, and covariance weighting. GNSS measurements contribute more strongly because of their smaller residual variances, while eLoran primarily improves measurement redundancy and geometric diversity.

**Figure 6:**
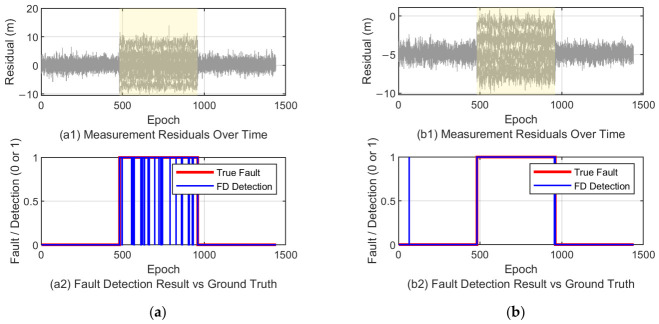
Residual and binary fault-detection results for (**a**) the 3-satellite + 3-eLoran configuration and (**b**) the 5-satellite + 1-eLoran configuration.

Figure 6 further compares the two reduced-observation configurations. The residual curves show clear deviations during the fault interval, while the binary detection results confirm that the configuration containing more GNSS satellites generally achieves better detection stability than the configuration relying more heavily on eLoran measurements.

### 4.2. MDB and Elevation-Angle Effects

**Figure 7:**
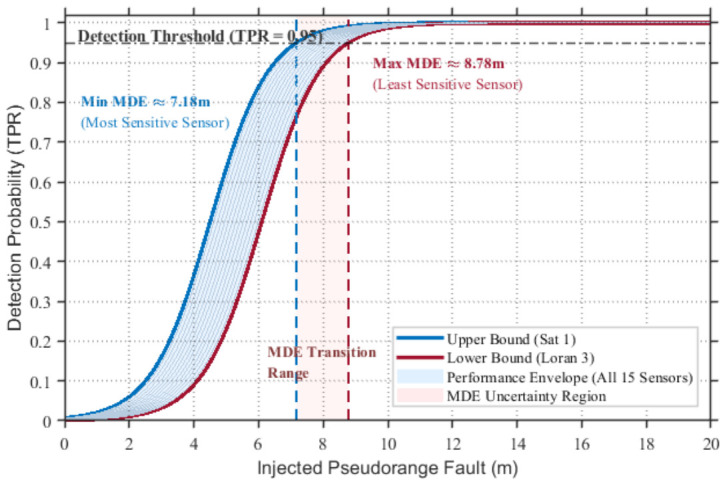
MDB values for BDS satellites under BDS/eLoran hybrid geometry.

The MDB was further analyzed to quantify the sensitivity of the proposed hybrid integrity-monitoring model. Figure 7 shows the MDB values for BDS satellites under the BDS/eLoran hybrid geometry. The results indicate that when an 8 m fault was injected, all satellites could be detected with a true positive rate of 0.95. This demonstrates that the integration of eLoran measurements improves the fault-detection boundary and enhances the detectability of satellite measurement anomalies. The false positive rate is defined as the percentage of fault-free epochs that are incorrectly classified as fault epochs after the fault exclusion procedure. The 95% confidence level was selected for algorithm validation and comparative performance evaluation rather than for certification-level aviation integrity applications.

**Figure 8:**
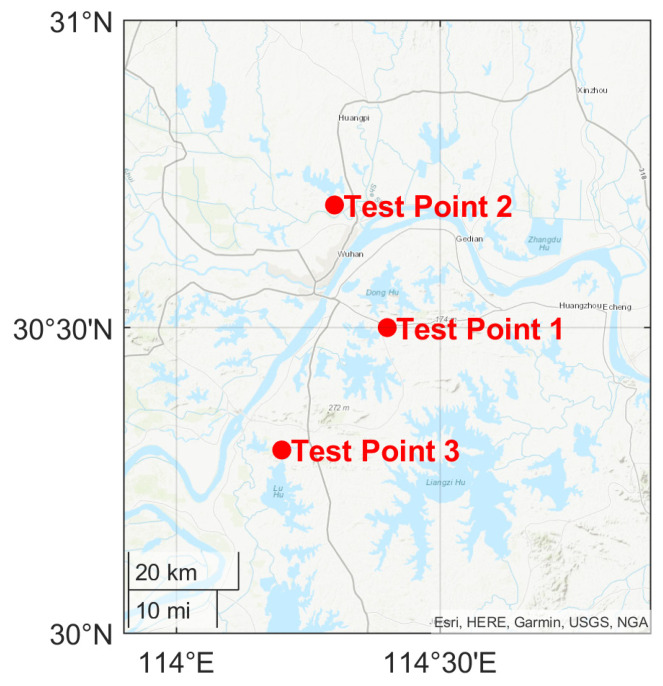
Locations of the three test points near Wuhan.

To evaluate the spatial robustness of the proposed method, three test points near Wuhan were selected, as shown in Figure 8. The corresponding detection results are summarized in Table 3.

The fault-detection results at the three test points are summarized in Table 3. The 12-satellite + 3-eLoran configuration provides the best overall performance, achieving essentially complete detection of step faults and consistently high detection rates for ramp faults. Under the severely satellite-deficient 3-satellite + 3-eLoran configuration, the detection rates remain around 90%, demonstrating that eLoran measurements can preserve useful integrity-monitoring capability when conventional GNSS-only RAIM lacks sufficient redundancy. The 5-satellite + 1-eLoran configuration generally outperforms the 3-satellite + 3-eLoran configuration, despite having the same total number of observations. This comparison further confirms that GNSS measurement quality and geometry have a stronger influence on detection stability, while eLoran serves mainly as a complementary source of redundancy. Ramp faults are slightly more difficult to detect than step faults because their initial magnitude is less distinguishable from nominal residual variations.

**Figure 9:**
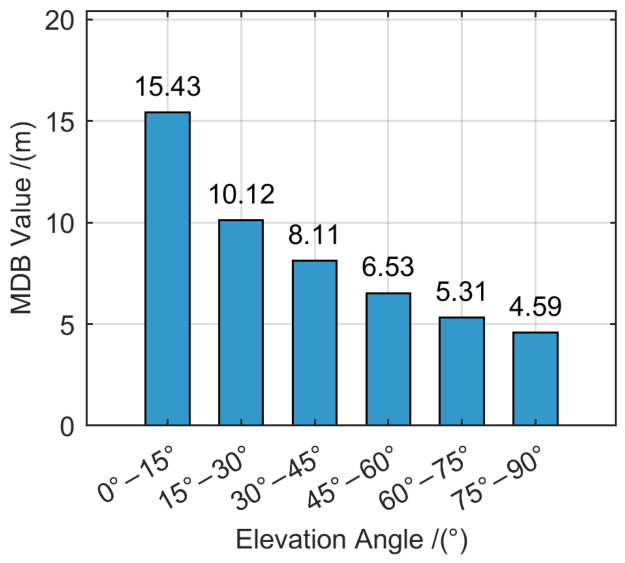
Average MDB of satellite SAT3 across different elevation intervals.

Finally, the influence of satellite elevation angle was investigated. The elevation angles of the inclined geosynchronous orbit (IGSO) satellite SAT3 observed at station WUH2 were divided into 15° intervals within the range of 0–90°, and the average MDB values were calculated for each interval. As shown in Figure 9, the MDB decreases as the satellite elevation angle increases, indicating that higher-elevation satellites are associated with better fault detectability. This result is consistent with classical RAIM theory, since low-elevation satellites are more strongly affected by atmospheric delay, multipath, and weaker geometric leverage.

**Figure 10:**
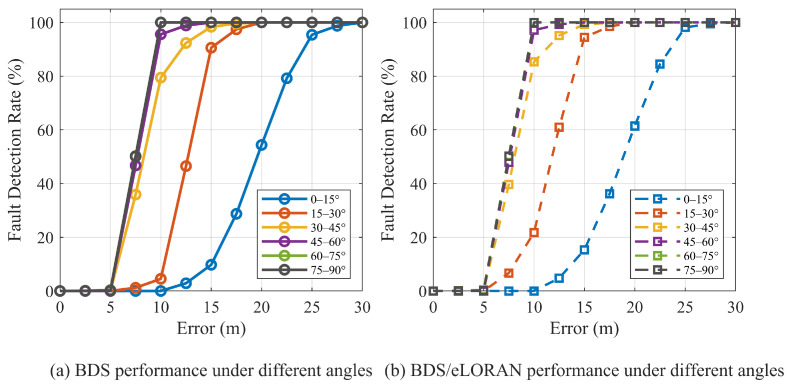
Fault detection performance of BDS and BDS/eLoran fusion for satellite SAT3.

Figure 9 and Figure 10 jointly illustrate the effects of satellite elevation angle and injected fault magnitude on fault-detection sensitivity. As shown in Figure 9, the average minimum detectable bias decreases with increasing satellite elevation, indicating that higher-elevation satellites provide better fault observability because they are less affected by atmospheric delay, multipath, and weak geometric leverage. Figure 10 further shows that the detection probability increases monotonically with fault magnitude and follows a clear sigmoid-like pattern, consisting of an initial low-sensitivity region, a rapid transition region, and a final saturation region close to 100%.

For a given fault magnitude, higher-elevation intervals generally reach high detection probabilities earlier than lower-elevation intervals. Moreover, the BDS/eLoran fusion curves are consistently above the corresponding BDS-only curves in the transition region. This indicates that the addition of eLoran enables the detector to reach the same detection probability at a smaller fault magnitude. The improvement is particularly relevant for lower-elevation satellites, where the BDS-only geometry is weaker, confirming that eLoran integration enhances measurement redundancy and overall fault-detection sensitivity.

**Figure 11:**
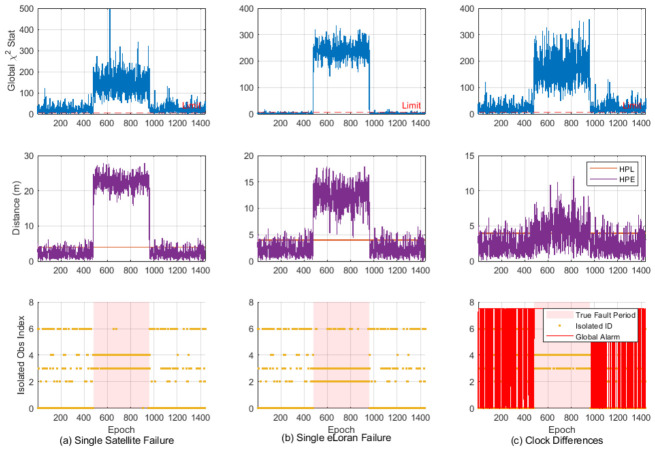
Integrity-monitoring results under (**a**) a single-satellite step fault, (**b**) a single-eLoran-station step fault, and (**c**) a GNSS–eLoran clock synchronization bias. The rows show the global test statistic, HPL/HPE comparison, and fault-detection or integrity-alert results, respectively.

To evaluate the integrity performance of the proposed GNSS/eLoran hybrid RAIM framework, three representative fault scenarios were investigated over 1440 epochs, including a single-satellite fault, a single eLoran station fault, and a GNSS–eLoran clock synchronization bias. Figure 11 demonstrates that the system maintains consistent integrity assurance under both localized and systemic fault conditions.

For localized faults (Scenarios 1 and 2), the global $\chi^{2}$ statistic rapidly exceeds the detection threshold during the fault interval (epochs 480–960), enabling effective fault detection and exclusion. In both cases, the subset separation mechanism successfully identifies and removes the faulty measurement, thereby preserving navigation continuity. As a result, the HPE remains well below the HPL, confirming the effectiveness of fault isolation in both GNSS and eLoran channels.

For the systemic clock bias scenario (Scenario 3), the fault violates the single-measurement assumption, preventing precise fault isolation. Instead, the global test triggers continuous integrity alerts, while the protection level adaptively increases to account for elevated system uncertainty. This conservative response ensures that HPE remains bounded by HPL, thereby preventing hazardous misleading information under common-mode failure conditions.

Overall, the results verify that the proposed hybrid RAIM framework provides robust integrity assurance across both isolated and system-wide fault modes, achieving either fault exclusion or timely alerting to maintain bounded positioning error.

## 5. Discussion

The results presented in Section 4 demonstrate that the principal benefit of GNSS/eLoran integration is the preservation of integrity-monitoring capability when GNSS measurement redundancy becomes insufficient. Conventional GNSS-only RAIM requires a minimum number of independent observations for fault detection and exclusion. When satellite visibility is severely degraded, this redundancy condition may no longer be satisfied. By introducing terrestrial eLoran measurements, the proposed framework supplements the observation set and enables the global consistency test and solution-separation procedure to remain operational under satellite-deficient conditions. Therefore, the primary contribution of eLoran is not to replace high-accuracy GNSS measurements, but to reduce the occurrence of integrity-monitoring gaps by providing additional ranging information from an independent physical infrastructure.

The comparison among the tested observation configurations further shows that measurement number alone does not determine integrity performance. The statistical contribution of each observation is jointly governed by its geometry and assigned covariance. GNSS pseudoranges generally have smaller residual variances and consequently receive larger weights in the weighted least-squares solution, whereas eLoran observations contain larger propagation-dependent uncertainties, particularly those associated with residual ASF errors and inter-system timing uncertainty. This explains why the configuration containing more GNSS satellites generally provides more stable detection performance than a configuration with the same total number of measurements but a larger proportion of eLoran observations. Nevertheless, when GNSS redundancy is insufficient, eLoran still provides valuable geometric diversity and fault observability that would otherwise be unavailable. The results, therefore, indicate a complementary relationship: GNSS measurements dominate estimation accuracy and detection stability, while eLoran measurements primarily improve redundancy, continuity, and availability.

The different responses to step and ramp faults are also consistent with the operating principle of the residual-based detector. A step fault produces an immediate inconsistency in the measurement set and, therefore, causes the global test statistic to cross the detection threshold rapidly. In contrast, a ramp fault initially remains close to the nominal noise level and becomes detectable only after the accumulated bias is sufficiently large. The lower early-stage sensitivity to ramp faults is, therefore, not specific to eLoran integration, but is an inherent characteristic of snapshot residual testing. Even so, the experiments show that the hybrid framework can detect both abrupt and gradually developing anomalies once their influence becomes statistically distinguishable from the modeled measurement uncertainty. The minimum-detectable-fault and elevation-angle results additionally confirm that detection sensitivity depends on satellite geometry: measurements with stronger geometric contribution generally produce larger residual responses for the same injected fault magnitude.

The integrity results also clarify the distinction between fault exclusion and conservative alerting. Under a localized single-measurement fault, the solution-separation procedure can identify the inconsistent GNSS or eLoran observation and recompute the navigation solution after exclusion. Under a common inter-system clock-bias disturbance, however, several eLoran observations may be affected simultaneously, violating the single-fault assumption adopted by the current implementation. In this case, reliable isolation is not guaranteed. The appropriate integrity response is, therefore, to issue an alert and enlarge the protection level rather than incorrectly attribute the disturbance to one measurement. The fact that the Horizontal Position Error remains bounded by the Horizontal Protection Level in the tested cases indicates that the framework behaves conservatively when the assumed fault model is not fully satisfied.

Several limitations should be considered when interpreting these findings. First, the implemented fault hypothesis set is mainly based on independent single-measurement faults. Simultaneous satellite and eLoran faults, intermittent faults, transmitter-level failures, and spatially distributed ASF anomalies require an expanded multiple-hypothesis model. Second, the present eLoran covariance model uses variance inflation to represent residual propagation and synchronization uncertainties. Although this treatment prevents excessive weighting of eLoran observations, it does not explicitly model temporal variation or spatial correlation among ASF residuals. Incorporating adaptive covariance estimation and regional ASF correlation models could improve both detection sensitivity and protection-level reliability. Third, the evaluation is based on representative stations and simulated fault injection within the Asia–Pacific region. Further validation using real eLoran measurements, longer observation periods, additional geographic areas, and diverse propagation environments is required before operational deployment.

## 6. Conclusions

This study proposed a GNSS/eLoran hybrid integrity-monitoring framework for satellite-deficient environments. GNSS pseudoranges and eLoran range-equivalent observations were integrated using covariance-weighted least-squares estimation, a global chi-square consistency test, and solution-separation-based fault exclusion. The framework preserves the dominant accuracy contribution of GNSS while using eLoran to provide additional redundancy and geometric diversity.

The experiments showed that the proposed method achieved strong detection performance under high-redundancy conditions and maintained practical integrity-monitoring capability when the available GNSS measurements were insufficient for conventional RAIM. Step faults were detected more rapidly than ramp faults, and configurations containing a larger proportion of GNSS measurements generally exhibited greater stability. The elevation-angle and fault-magnitude analyses further showed that eLoran integration improves detection sensitivity, particularly under weak GNSS geometry. In all tested integrity scenarios, the horizontal position error remained bounded by the protection level.

Future work will extend the present single-measurement fault model to simultaneous and transmitter-level faults, incorporate adaptive and spatially correlated ASF covariance models, and validate the framework using real eLoran observations in broader propagation environments.

## Figures and Tables

**Table 1:** Comparison with existing methods.

Method	Measurement Type	Fault Model	Complexity	Integrity Capability
Classical RAIM	GNSS only	single fault	low	limited (RAIM holes)
ARAIM	GNSS only	multi-hypothesis	high	high but satellite-dependent
GNSS/INS RAIM	GNSS + inertial	sensor fusion	medium-high	improved continuity
Proposed method	GNSS + eLoran	heterogeneous faults	medium	robust in satellite-deficient environment

**Table 2:** Experimental configuration for the GNSS/eLoran hybrid RAIM simulation.

Item	Configuration	Description
Satellite selection	Geometric dilution of precision (GDOP) based optimization	Satellite subsets are selected by minimizing GDOP to ensure favorable spatial geometry and improved dilution of precision.
eLoran stations	East China Sea chain (China coastal system)	A regional terrestrial LORAN chain deployed in the eastern coastal region of China is used to provide complementary ranging observations.
Measurement noise model	System- and propagation-aware stochastic model	GNSS measurement noise includes receiver noise, multipath, and residual atmospheric delays. eLoran noise accounts for receiver thermal noise, transmitter timing uncertainty, and propagation-dependent errors including ASF residuals and terrain/ground-conductivity-induced variations.
Step fault model	20 m bias injection	A constant step-like pseudorange bias of approximately 20 m is introduced into selected measurements to simulate abrupt hardware or signal anomalies.
Ramp fault model	0.05 m/s drift rate	A linearly increasing pseudorange error with a slope of 0.05 m/s is applied to model slowly developing faults.

**Table 3:** Fault-detection performance of the proposed hybrid RAIM algorithm at different test points.

Error Type	Test Points	System Combination Type	Fault Detection Rate (%)	False Alarm Rate (%)	False Positive Rate (%)
Step Fault	1	12 satellites + 3 eLoran	100	0	0
		3 satellites + 3 eLoran	90.23	5.63	3.75
		5 satellites + 1 eLoran	100	0	0
	2	12 satellites + 3 eLoran	100	0	0
		3 satellites + 3 eLoran	91.45	5.79	4.01
		5 satellites + 1 eLoran	100	0	0
	3	12 satellites + 3 eLoran	100	0	0
		3 satellites + 3 eLoran	89.94	5.12	4.36
		5 satellites + 1 eLoran	98.23	1.05	0.97
Ramp Fault	1	12 satellites + 3 eLoran	97.52	0.95	0.97
		3 satellites + 3 eLoran	92.10	1.97	1.25
		5 satellites + 1 eLoran	95.53	1.35	1.22
	2	12 satellites + 3 eLoran	96.94	1.02	0.99
		3 satellites + 3 eLoran	90.26	2.11	1.47
		5 satellites + 1 eLoran	94.59	1.37	1.24
	3	12 satellites + 3 eLoran	98.13	0.96	0.84
		3 satellites + 3 eLoran	91.47	5.12	4.36
		5 satellites + 1 eLoran	98.23	1.05	0.97

## Data Availability

The raw data supporting the conclusions of this article will be made available by the authors on request.
